# A novel computational method for automatic segmentation, quantification and comparative analysis of immunohistochemically labeled tissue sections

**DOI:** 10.1186/s12859-018-2302-3

**Published:** 2018-10-15

**Authors:** Elena Casiraghi, Veronica Huber, Marco Frasca, Mara Cossa, Matteo Tozzi, Licia Rivoltini, Biagio Eugenio Leone, Antonello Villa, Barbara Vergani

**Affiliations:** 1 Department of Computer Science “Giovanni Degli Antoni”, Università degli Studi di Milano, Via Celoria 18, 20135 Milan, Italy; 2 Unit of Immunotherapy of Human Tumors, Department of Experimental Oncology and Molecular Medicine, Fondazione IRCCS Istituto Nazionale dei Tumori, Milan, Italy; 3 Department of medicine and surgery, Vascular Surgery, University of Insubria Hospital, Varese, Italy; 4 School of Medicine and Surgery, University of Milano Bicocca, Monza, Italy; 5 Consorzio MIA – Microscopy and Image Analysis, University of Milano Bicocca, Monza, Italy

**Keywords:** Histochemical and immunohistochemical image analysis, Digital image processing, Statistical analysis, Supervised learning methods, Comparative analysis

## Abstract

**Background:**

In the clinical practice, the objective quantification of histological results is essential not only to define objective and well-established protocols for diagnosis, treatment, and assessment, but also to ameliorate disease comprehension.

**Software:**

The software MIAQuant_Learn presented in this work segments, quantifies and analyzes markers in histochemical and immunohistochemical images obtained by different biological procedures and imaging tools. MIAQuant_Learn employs supervised learning techniques to customize the marker segmentation process with respect to any marker color appearance. Our software expresses the location of the segmented markers with respect to regions of interest by mean-distance histograms, which are numerically compared by measuring their intersection. When contiguous tissue sections stained by different markers are available, MIAQuant_Learn aligns them and overlaps the segmented markers in a unique image enabling a visual comparative analysis of the spatial distribution of each marker (markers’ relative location). Additionally, it computes novel measures of markers’ co-existence in tissue volumes depending on their density.

**Conclusions:**

Applications of MIAQuant_Learn in clinical research studies have proven its effectiveness as a fast and efficient tool for the automatic extraction, quantification and analysis of histological sections. It is robust with respect to several deficits caused by image acquisition systems and produces objective and reproducible results. Thanks to its flexibility, MIAQuant_Learn represents an important tool to be exploited in basic research where needs are constantly changing.

## Background

Over the past decades, continuous increase in computational power, together with substantial advance in digital image processing and pattern recognition fields, have motivated the development of computer-aided diagnostic (CAD) systems. Thanks to their effective, precise and repeatable results, validated CAD systems are nowadays exploited as a valid aid during diagnostic procedures. [1–5]. With the advent of high-resolution digital images, the development of computerized systems helping pathologists during analysis of images obtained by histochemical (HC) and immunohistochemical (IHC) labeling has become a main research focus in microscopy image analysis.

State-of-the-art automatic image analysis systems automatically identify (segment) markers (stained areas), and then try to reproduce the evaluation and quantification performed by expert pathologists [6–9]. These tools could have the potential to minimize the inherent subjectivity of manual analysis and to largely reduce the workload of pathologists via high-throughput analysis [10–12].

Generally, after color transformation, illumination normalization, color normalization and noise reduction, the current methods firstly compute a rough marker segmentation, refine the detected structures, and finally quantify them. Noise reduction is performed by applying median filters [13, 14], Gaussian filters [15] and morphological gray-scale reconstruction operators [16]. Attention is devoted to the color transformation process, which should overcome the problematic and undesirable color variation due to differences in color responses of slide scanners, raw materials and manufacturing techniques of stain vendors, as well as staining protocols across different pathology labs [17]. While some systems transform the RGB color space into more perceptual color spaces, such as CIE-Lab [18–22], Luv [23–25], Ycbcr [26], or 1D/2D color spaces [18, 27, 28], others perform illumination and color normalization through white shading correction methods [29, 30], background subtraction techniques, (adaptive) histogram equalization [14, 31, 32], Gamma correction methods [33], Reinhard’s method [34], (improved) color deconvolution [35, 36], Non-negative Matrix Factorization (NMF) and Independent Component Analysis (ICA) [17, 37–40], decorrelation stretching techniques [14, 32, 41], anisotropic diffusion [22]. After these preprocessing steps, the labeled structures of interest are detected by morphological binary or gray level operators [28, 42–45], automatic thresholding techniques [20, 28, 33, 43], clustering techniques [46, 47], the Fast Radial Symmetry Transform (FRST) [16, 48], Gaussian Mixture Models [20, 22, 49], and edge detectors such as the Canny edge detector, Laplacian of Gaussian filters [50] or Difference of Gaussian filters [51]. These algorithms are followed by techniques that refine the extracted areas, through methods such as the Hough transform [51], Watershed algorithms [45, 52–54], Active Contour Models [45, 51, 55], Chan-Vese Active Contours [54, 56], region growing techniques [19], different graphs methods [57], or graph-cuts segmentation techniques [50, 58]. Extracted areas can be also refined by more complex learning techniques such as rule based systems [59], cascades of decision tree classifiers [60], Bayesian Classifiers [42], KNN classifier [61] trained on RGB color coordinates [59], the Quadratic Gaussian classifier [43, 62], Convolutional Neural Networks (CNN) [63] or SVMs [51]. Marker quantification methods vary a lot, depending on the clinical research question and the required tasks.

The lack of flexibility with respect to different image characteristics, whose variability depends on the acquisition system and the specific staining procedure used to dye the image, hampers the ample application of state of the art automatic histological image analysis systems. Image problems, depending on the presence of tissue folds and/or cuts, unspecific colorations and unwanted background structures, additionally misguide the image analysis systems. Though some effective methods exist, given the high image resolutions and dimensions, their usage of particularly complex image processing techniques often makes them too expensive in terms of computational time and memory storage.

However, automatic analysis is increasingly demanded for its objective, precise and repeatable numerical estimates on a statistically significant number of high-resolution images. Our open source software MIAQuant [59] effectively segments and quantifies markers with specific colorings from histological images, by combining simple and efficient image processing techniques. Upon providing contiguous (serialized) tissue sections, MIAQuant aligns them and computes an image where the markers are overlapped with different colors, thus allowing the visual comparison of the markers’ respective locations. Its effective results in biomedicine motivated us to expand its ability to express the localization of markers stained on different, and eventually contiguous serialized, tissue sections. Similar to MIAQuant, our improved system called MIAQuant_Learn exploits simple, efficient, and effective image processing, pattern recognition and supervised learning techniques [64], with the aim of customizing the marker segmentation to any color appearance. MIAQuant_Learn computes mean-distance histograms to objectively express the markers’ position and relative location with respect to the resection margins and to user-selected structures of interest. In case of serial tissue sections, MIAQuant_Learn computes objective “morphology-based” measures expressing the markers’ co-existence in areas of higher densities.

## Implementation

**Fig. 1:**
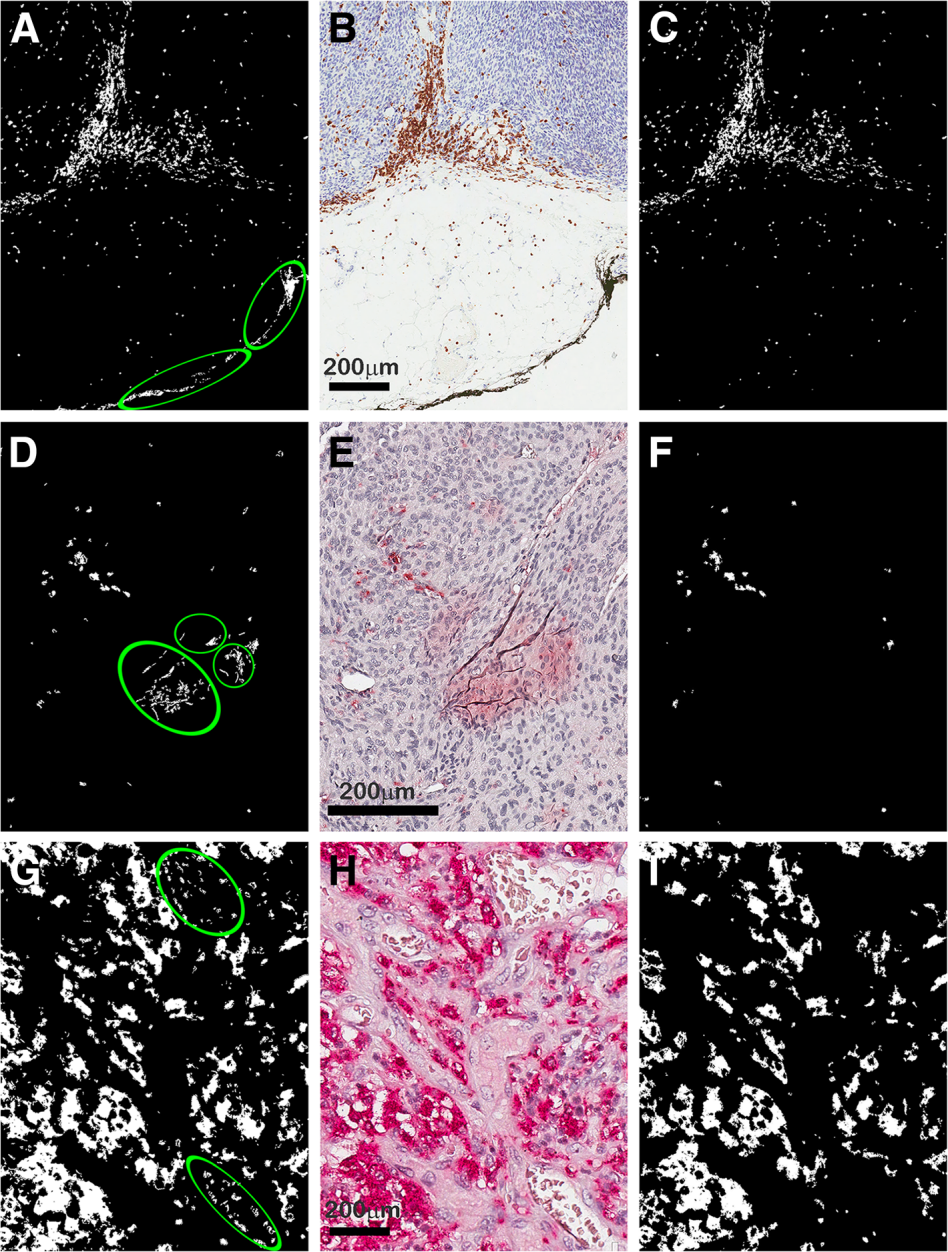
Comparison of segmentation results obtained by MIAQuant and MIAQuant_Learn on critical images. Center column: sample sub-images containing CD3 (**b**) markers stained in brownish color, and CD163 (**e** and **h**) markers stained in reddish color. The images contain unwanted ink deposits (**b**), and unwanted background (**e** and **h**). Left column: marker segmentation results produced by MIAQuant (**a**, **d** and **g**) contain false positives pixels belonging to ink (**a**) and unwanted background (**d**, **g**). Right column: marker segmentation results produced by MIAQuant_Learn (**c**, **f** and **i**) contain only true positive pixels

MIAQuant_Learn is an improved version of MIAQuant, developed to overcome MIAQuant’s main limits and expand its capabilities. The extensive usage of MIAQuant has evidenced lack of robustness, with respect to both imaging system related artifacts, and specific problems arising during procedures such as tissue preparation and staining. Some examples are detailed in Fig. 1, showing sub-images containing unspecific colorings (center-column), which are wrongly included by MIAQuant’s segmentation results (left column), since their color appearance is too similar to that of markers. Another drawback of MIAQuant relies in the fact that it allows segmenting only markers whose color appearance is coded into the rule-based system. However, the user might need to expand the segmentation capabilities, to extract markers whose color appearance differs from that actually recognized by MIAQuant.

MIAQuant_Learn is a prototype software developed with Matlab R2017a on a standard laptop (CPU: Intel i7, RAM 16 GB, disk 256 SSD). The system requirements depend on the image size and resolutions; based on our memory storage limits, MIAQuant_Learn is able to open and process images stored with lossless compression techniques (e.g. image formats TIFF, JPEG 2000, or PNG), provided their memory size is less than 2 GB (images whose pixel dimension is about 25000 × 25000). To circumvent this limit, before processing, we vertically slice top weight images (by a Linux script); MIAQuant_Learn processes each slice and recompose the computed results upon analysis. To fasten the algorithms, we developed MIAQuant_Learn by exploiting the parallel computing Toolbox provided by Matlab R2017a, which allows solving computationally and data-intensive problems using multicore processors without CUDA or MPI programming. In detail, the toolbox lets the programmer use the full processing power of multicore desktops by programming applications that are distributed and executed on parallel workers (MATLAB computational engines) that run locally.

**Fig. 2:**
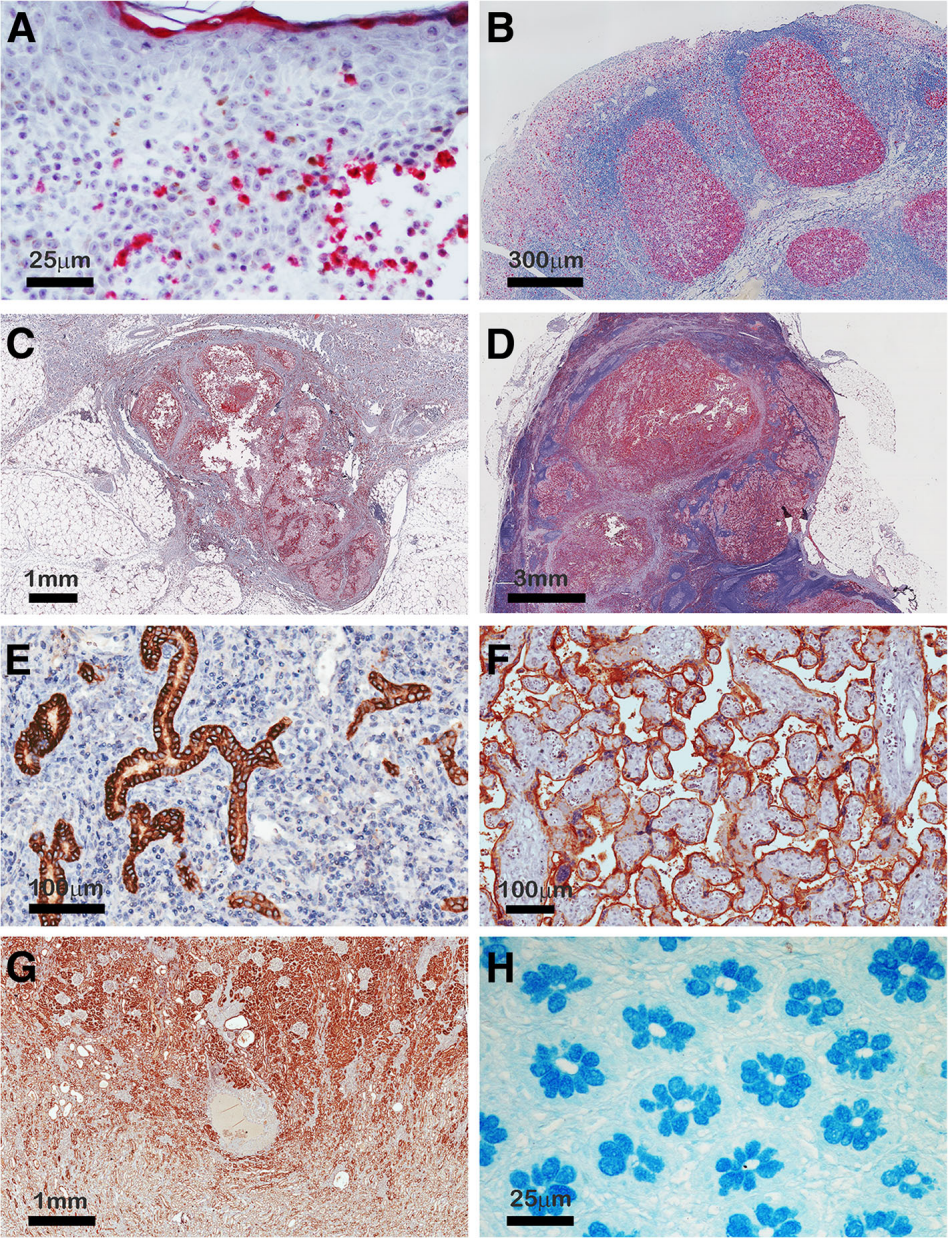
Immunohistochemically stained human sections showing the high color and texture variability characterizing histological images. **a** pyoderma gangrenosum marked with arginase antibody (Arg1); **b** human tonsil marked with Ki-67 antibody; **c** subcutaneous metastatic melanoma marked with CD163 antibody; **d** lymph node metastatic melanoma marked with CD163 antibody **e** liver cirrhosis marked with carbonic anhydrase IX antibody; **f** placenta marked with PDL-1 antibody; **g** kidney marked with V-ATPase H1 antibody; **h** colon marked with alcian blue. Thanks to the usage of supervised learning techniques, MIAQuant_Learn can be customized to effectively segment markers characterized by different stains and textures

MIAQuant_Learn has been developed and tested on digital (RGB color) HC and IHC images representing different tissue samples acquired by different imaging systems (e.g.: Aperio Scanscope Cs, Olympus BX63 equipped with DP89 camera and software cellSens, or Nikon Eclipse E600 microscope equipped with DS-Fi1 camera and software Nis-Elements AR3.10). Up to now, MIAQuant_Learn has processed 1357 RGB images belonging to 11 different datasets (sample tissue sections stained with different colors are shown in are shown in Fig. 2), each containing “biologically” similar (pathological and/or healthy) tissue samples. Each dataset is composed of images with a specific image resolution (resolution range [0.4 η/px - 8 η/px]). When the considered dataset contains serialized section sets, each set is generally composed of 3 to 7 serial IHC-stained sections to visualize different markers and the processed images are characterized by a high pixel dimension (ranging from 15000x15000x3 px to 35000x35000x3 px).

Importantly, MIAQuant_Learn avoids any preprocessing step for noise reduction, illumination normalization, and color normalization, since our experimental results have shown that these procedures might excessively alter, or even delete, small marker areas (which will be simply referred as markers). In the following, the main steps of MIAQuant_Learn are described.

### Segmentation of the tissue region

**Fig. 3:**
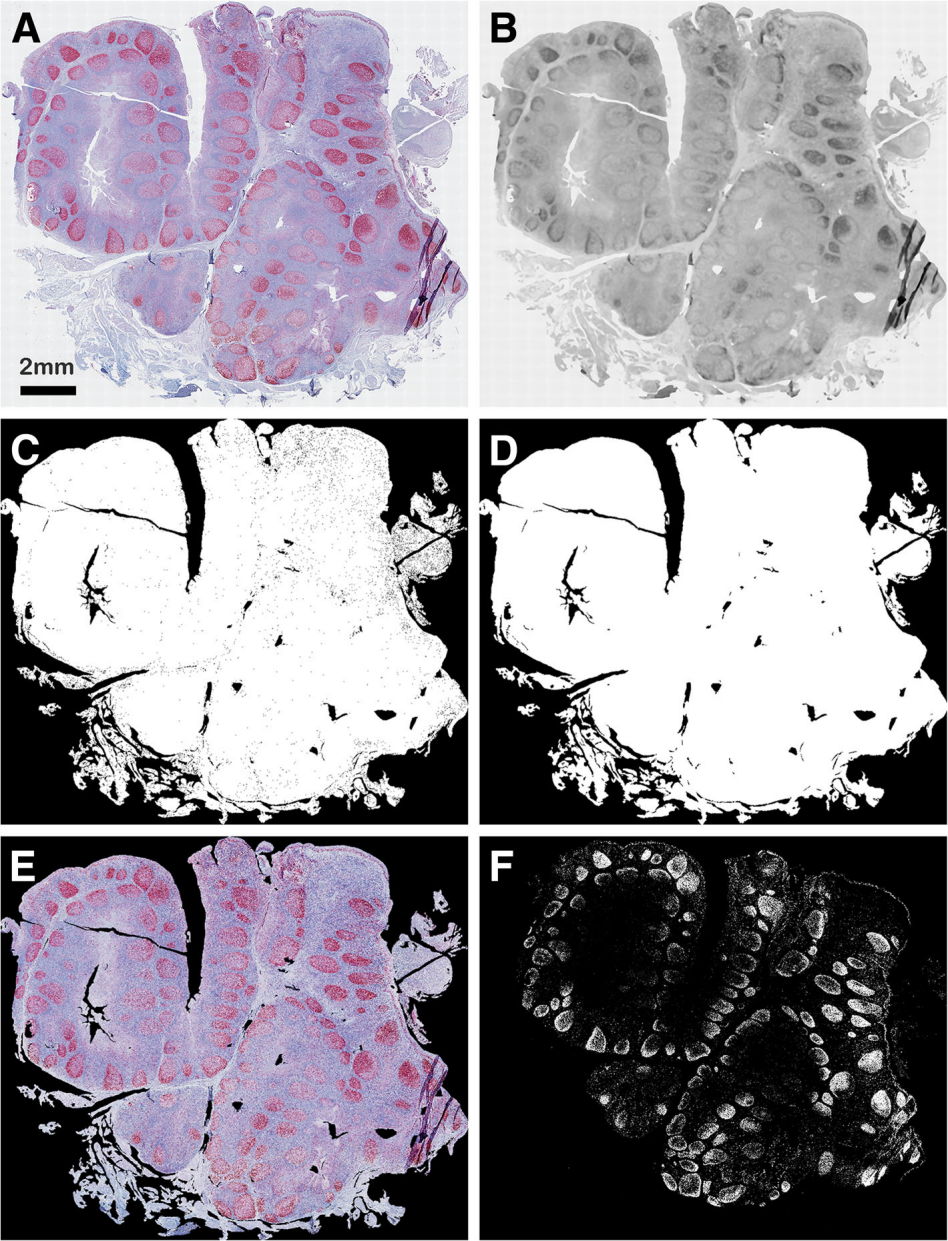
Steps of the tissue region segmentation procedure. **a** original image depicting human tonsil marked Ki-67 antibody; **b** gray level image after the heavy filtering process; **c** segmented tissue region before its refinement by applying morphological operators; **d** segmented tissue region after morphological “cleaning” and holes filling; **e** RGB image included in the tissue region; **f** segmented markers

Firstly, the tissue region is extracted to restrict the processing region. To this aim, the image is downsampled (to avoid high computational costs) to a size less or equal to 5000 pixels, it is transformed into its gray-level (*gL*) version [13] and filtered with a 25 × 25 px median filter followed by a Gaussian filter with standard deviation equal to 0.5. This heavy filtering process allows to abruptly reducing salt-and-pepper and Gaussian noise, creating a smoothed image where an (almost) uniform brighter background is contrasted with the darker tissue region. The resulting tissue mask, distinguishable from the background by automatically thresholding the filtered image with the Otsu algorithm [65], is then rescaled to the original image size and is refined to remove false positive segmentation errors (pixels wrongly included in the tissue mask originating from scale reduction and filtering process). These pixels are in the border of the tissue mask and can be recognized by their bright *gL* value, which is similar to that of background pixels. To detect false positive pixels, we therefore compute the mean (*mean*_*back*_) and the standard deviation (*std*_*back*_) of the *gL* values of pixels included into the background, and we remove from the tissue mask those pixels *p* such that: *gL*(*p*) > *mean*_*back*_ + 0.5 ∗ *std*_*back*_. The obtained mask is further refined by filling small holes [13] and by removing connected areas that are speculated, not compact, or too small. Finally, to reduce the memory storage requirements, the image is cropped to strictly contain the tissue region. Figure 3 shows the results computed by the main steps of the tissue-region segmentation procedure. Note that, though the segmentation result might be quite rough, the applied simple processing steps effectively allow to restrict the processing area without requiring too much computational time.

Since manual segmentations performed by experts were not available, to straightforwardly assess the tissue-region segmentation step we showed 500 images to three experts and asked them to assign the following grades: A (perfect segmentation), B (small presence of false positives and/or false negative errors), C (evident presence of false positive and/or false negative errors), D (bad segmentation). Overall, 487 images were assigned grade A (97.4%), 10 images grade B (2%), while 3 of them contained evident errors and were graded with C (0.6%). This visual analysis has demonstrated the effectiveness of the tissue-region segmentation step.

### Marker segmentation via decision trees, support vector machines, and K-nearest neighbor

To let the user customize MIAQuant_Learn to segment any marker colorings, we employ a simple stacked classifier, which combines the results obtained by decision trees (*DTs*), support vector machines (*SVMs*) with radial basis function kernels, and one K-Nearest Neighbor (*KNN*) classifier.

For computational efficiency MIAQuant_Learn avoids the usage of classifiers requiring high computational costs and memory storage, such as deep learners (e.g.: deep neural networks, convolutional neural networks, deep belief networks, deep recurrent neural networks), nowadays widely used in the medical image analysis research field [66, 67]. Additionally, since any image transformation is time-consuming, we characterize each pixel with a small set of RGB color features computed over its 7 × 7-neighborhood, and we avoid more complex texture features (e.g.: entropy, derivatives, Fourier descriptors [68]). This strategy allows splitting big images into smaller sub-images, separately processing them, and recomposing the obtained segmentations for further analysis.

In detail, given a pixel *p*, and being {*R*_*p*_, *G*_*p*_, *B*_*p*_} its RGB color coordinates,^1^ *p* is represented by the 24 dimensional feature vector: where: *μ*_*nRGB*_ = {*μ*_*nR*_, *μ*_*nG*_, *μ*_*nB*_} is a three dimensional vector containing the mean RGB color values of pixels in the *n-by-n*-neighborhood of *p*, the vector *σ*_*nRGB*_ = {*σ*_*nR*_, *σ*_*nG*_, *σ*_*nB*_} contains the standard deviations of the RGB color values of pixels in the *n-by-n*-neighborhood of *p*, while *range*_*nRGB*_ = {*range*_*nR*_, *range*_*nG*_, *range*_*nB*_} contains the local ranges (maximum-minimum RGB values) of the *n-by-n* neighborhood of *p*.

### Training data collection

To collect training data we developed a user interface showing sample sub images to experts with the following selection possibilities:

- “marker-pixels” (positive training samples), that is pixels belonging to markers.
- Rectangular areas containing only “not-marker pixels” (obvious negative training samples); these areas generally contain the most “obvious” not-marker-pixels and do not carry enough information to discard not-marker-pixels whose appearance is similar to that of marker-pixels.
- “Critical not-marker”-pixels (critical negative training samples); these are those not-marker pixels whose an appearance is very similar to marker-pixels.

**Fig. 4:**
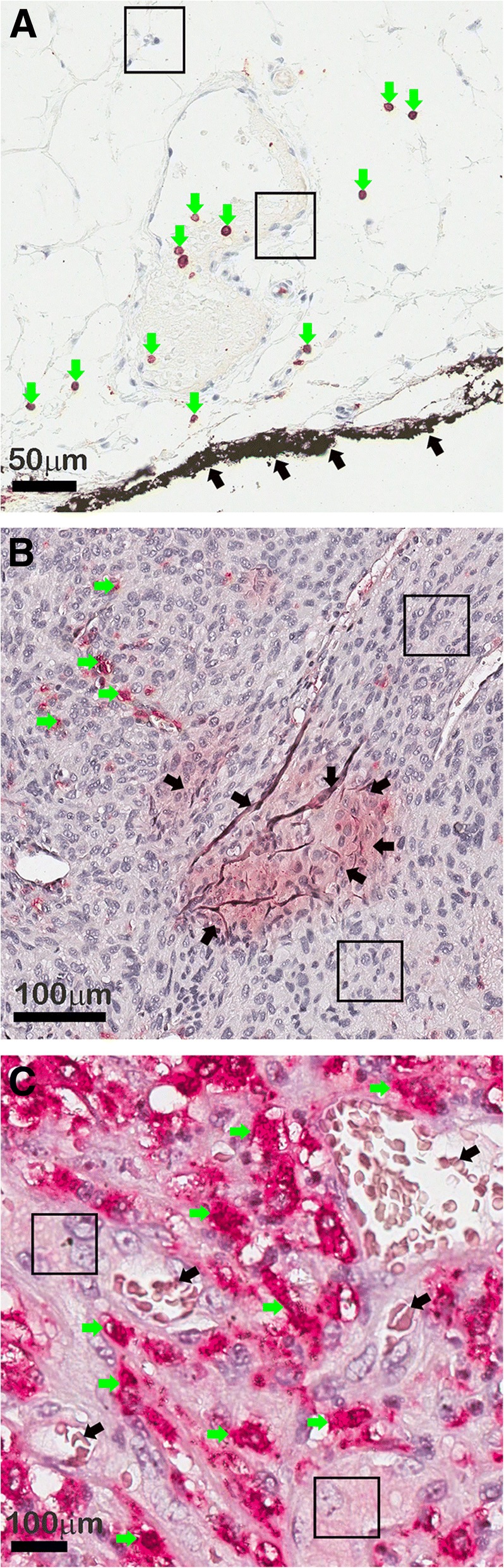
Selection of training pixels. **a** sample tissue images showing CD3 marker stained with brownish color (**a**), and CD163 marker stained in reddish color (**b** and **c**). In the images we show examples of manually selected marker-pixels (green arrows), rectangular areas containing obvious not-marker pixels (black rectangular areas), and critical not-marker pixels (black arrows)

Figure 4 shows some examples of marker-pixels (green arrows), critical not-marker-pixels (black arrows), and rectangular areas containing no markers (black rectangles). With the described selection system we often obtain highly unbalanced training sets, where the number of positive samples, *Npos*, which is generally similar to the number of critical negative samples, *Ncrit*, is much lower than the number of obvious negative samples, *Nneg*. As a result, it can occur that the ratio of positive versus negative training samples is such that: $\frac{Npos}{Nneg+ Ncrit}\le \frac{1}{50}$.

### Classifying system

**Fig. 5:**
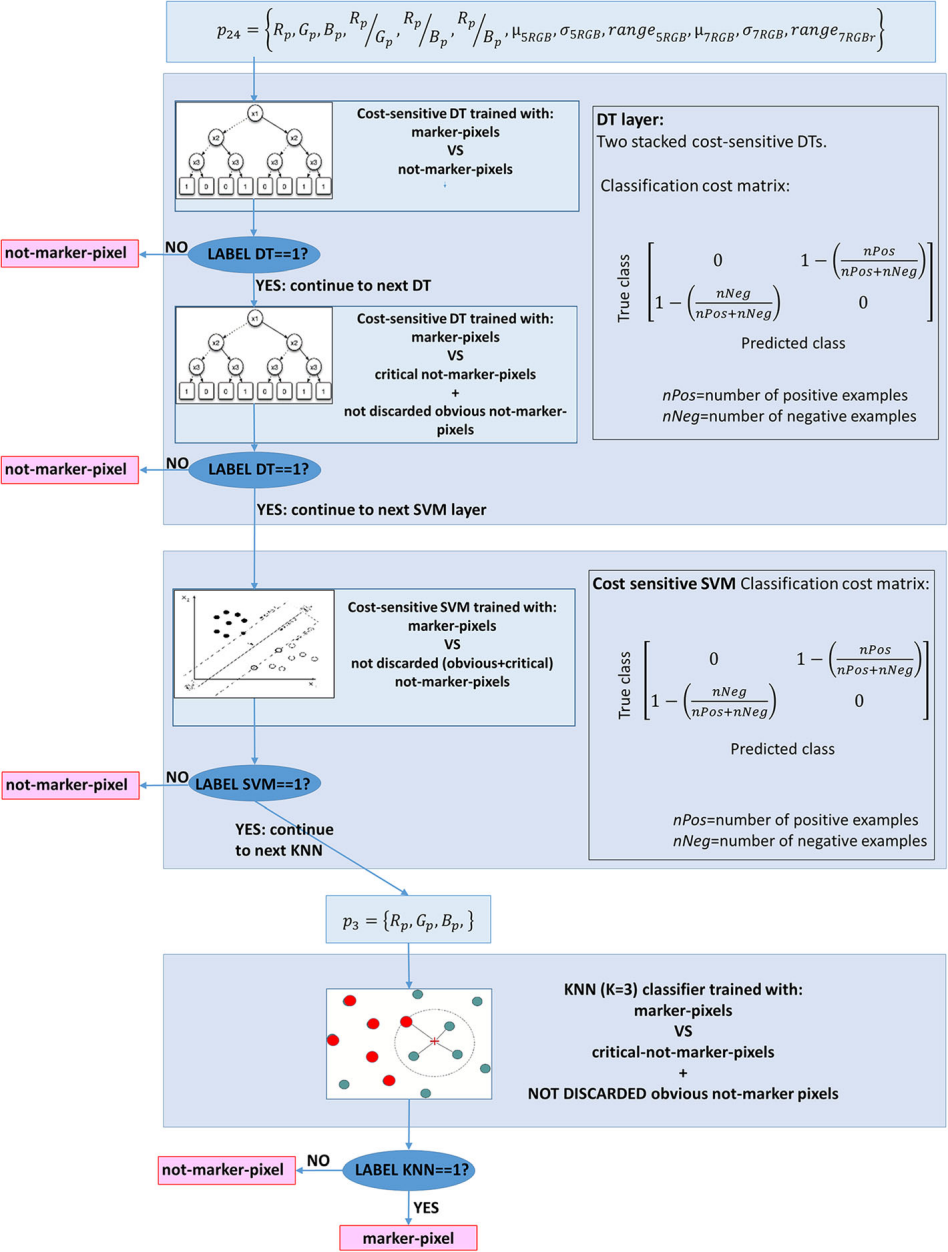
The structure of the stacked classifier

The stacked classifier, whose structure is schematized in Fig. 5, is composed by two stacked cost-sensitive decision trees (first DT layer), followed by one cost-sensitive SVM with radial basis function kernel (second SVM layer), followed by one KNN classifier (third KNN layer). Each classifier discards pixels recognized as not-marker pixels and leaves to the next classifiers any further decision regarding the pixels classified as (candidate) marker-pixels.

The misclassification cost of both the DTs and the SVM is

$$ Cost\left(p,t\right)=\left[\begin{array}{cc}0& 1-\frac{Nneg+ Ncrit}{Npos+ Nneg+ Ncrit}\\ {}1-\frac{Npos}{Npos+ Nneg+ Ncrit}& 0\end{array}\right] $$

where Cost(*p*, *t*) is the cost of classifying a point into class *p* if its true class is *t* (i.e., the rows correspond to the true class and the columns correspond to the predicted class). Label 1 is assigned to positive examples and label 0 is assigned to negative examples. This cost matrix assigns a higher misclassification cost to pixels belonging to the class whose training set has the lowest cardinality.

The KNN classifier is not cost-sensitive; it employs the cost matrix: $$ Cost\left(p,t\right)=\left[\begin{array}{cc}0& 1\\ {}1& 0\end{array}\right] $$.

While the DTs and the SVM are trained on the training pixels coded as 24 dimensional vectors, the KNN is trained on points *p* coded as 3-dimensional vectors *p*_3_ = {*R*_*p*_, *G*_*p*_, *B*_*p*_}.

The classifiers employ different training sets. The first DT is trained with an unbalanced training set composed of the training marker-pixels (positive examples) and all the training (obvious and critical) not-marker pixels (negative examples). The training points are coded as 24 dimensional vectors containing all the previously described features. 10-fold cross-validation is applied for training the first DT. Each fold is composed of $\frac{1}{10}\ast Npos$ randomly selected positive examples and $\min \left(\frac{1}{10}\ast \left( Nneg+ Ncrit\right),\kern0.5em 5\ast Npos\right)$ randomly selected negative examples; the remaining training pixels are used for validation. The trained DT that achieves the maximum accuracy is the chosen first DT classifier.

Once the first decision tree is trained, it is used to classify the set of obvious negative examples; after classification, only the wrongly classified samples (false positives) are kept as obvious negative training samples and added to the set of critical negative samples. The training set is therefore composed of all the positive examples, all the critical negative examples, and the wrongly classified negative examples. This process enormously reduces the number of available negative samples considered by the second DT, which is then trained by applying the aforementioned 10-fold cross validation to maximize the accuracy.

The second DT is then used to classify all the negative samples (critical + obvious) and only the wrongly classified negative examples are kept to train the following SVM classifier by applying 2-fold cross validation (to maximize the accuracy). The last layer is composed by one KNN classifier (with neighborhood size K = 3) working on points *p* coded as *p*_3_ = {*R*_*p*_, *G*_*p*_, *B*_*p*_}. It is trained on all the positive samples, all the critical negative samples, and the obvious negative samples wrongly classified by the preceding layers.

Applying the described stacked classifier we create a binary mask containing all the detected marker-pixels. This mask is “cleaned” by removing all connected components that have fewer than 3 pixels. These areas are too small to be considered and are often due to noise or image artifacts. The remaining connected areas are the extracted markers, whose quantification and comparative description is described in the following.

When applying the marker segmentation procedure to our database, after extracting some image samples, experts manually selected a training set of about 150 marker-pixels, 150 critical not-marker pixels and 15.000 obvious not-marker pixels (the selection of the obvious not-marker pixels, being based on rectangular selection areas, easily selects such a large number on negative examples). If some images were wrongly segmented (here it happened in 11% of 1357 images), the experts added extra training points by considering the wrongly segmented pixels. After retraining the classifiers and re-segmenting all the images in the dataset, we obtained remarkably good results for 98.63% of all images. Of note, when two datasets are “similarly stained”, that is they contain images whose markers have similar color appearances, the training procedure can be applied only once, since the marker-segmentation step can be performed by employing the same classifiers. Nonetheless, given a novel dataset to be segmented, the training set employed for a “similarly stained” dataset can be used as a starting training set, and extra training points can be added to obtain adequate classifiers. This allows to build semisupervised segmentation machines, easily adaptable with respect to different image datasets.

### Marker quantification and comparative measures for markers’ localization comparison

#### Mean-distance histograms from resection margins and structures of interest

Similar to MIAQuant, once marker segmentation has been applied on an input image, MIAQuant_Learn computes the marker density estimate as the percentage of the marker-pixels with respect to the tissue area (the tissue area is a scalar number, defined as the number of pixels in the tissue region). Precisely, given a section, *SL*, and denoting with *M* the markers segmented in *SL*, the density, *DM*_*T*_, of markers *M* in the tissue region of *SL* is computed as *DM*_*T*_ = *A*_*M*_/*TA* where *A*_*M*_ is the area covered by *M*, and *TA* is the tissue area in *SL*.

Additionally, MIAQuant_Learn expresses the marker location in the tissue region by computing normalized minimum-distance histograms estimating the distribution of the minimum distances^2^ between each marker-pixel and the borders of structures of interest, such as basement membrane, borders of cancer nodules, necrotic areas in plaques. When each marker is stained on a set of HC or IHC images, mean distance histograms can be computed for each marker. The visible similarities/dissimilarities of the mean distance-histograms computed for each marker objectively confirm the expected differences in the spatial distribution of the markers under analysis [69]. Indeed, experts consider the visualization of the distance-histograms as effective to understand the spatial distribution characterizing each marker. MIAQuant_Learn supports the visual comparison with a numerical measure: the difference among the normalized mean distance histograms of two markers, *M*_1_ and *M*_2_, is expressed by the average of the two histogram intersection measures (from *M*_1_ to *M*_2_, and from *M*_2_ to *M*_1_)^3^ [70].

#### Markers’ neighborhoods detection from sets of serial tissue sections

Given a set of serial sections, pathologists generally mark each to visualize the density and location of a specific structure, visually compare the labelled sections to find areas where the markers’ densities are high, and finally identify corresponding volumes where the analyzed markers (and hence the labelled structures) are mostly concentrated and neighboring.

MIAQuant_Learn provides means to help experts during this analysis.

Though contiguous, the sections we treat might have a quite different shape. Thus, when sets of marked serial tissue slices are available, MIAQuant and MIAQuant_Learn apply a multiscale-hierarchical registration procedure [59] to align the tissue masks as much as possible (tissue-shape registration).

Overall, we have employed this registration procedure on more than 40 sets of contiguous tissue sections (their cardinality varies in the range [3,…, 7]). To objectively evaluate the computed results, for each set composed of ***n*** serial tissue sections *{SL*_*1*_*, SL*_*2*_*,…, SL*_*n*_*}*, denoting with *T(SL*_*i*_*)* the tissue region in *SL*_*i*_, we define the **global tissue-region overlap** (***GTRO***), as: $GTRO=\frac{A\left({\bigcap}_{i=1}^nT\left({SL}_i\right)\right)}{A\left({\bigcup}_{i=1}^nT\left({SL}_i\right)\right)}\ast 100$, where *A*(*x*) is the numer of pixels of a binary region *x* For each set of serial tissue sections, we measured the *GTAO* before and after registration, and we computed the $mean(GTRO)=\frac{\sum_{j=1}^{40} GTRO(j)}{40}$ (where *GTRO*(*j*) is the *GTRO* computed for the j-th sets of serialized tissue sections). Before registration we measured a *mean*(*GTRO*) = 70.6 % (−7.2%, +8.3); after tissue shape registration this measure increased to a *mean*(*GTRO*) = 95.7 %  (−3.1%, +4.0%).

The registration step is followed by the computation of a color image where the different markers are shown with different colors, to allow an objective visual comparison of their relative location. MIAQuant_Learn also allows analyzing the aligned images, to numerically detect and express the co-existence (or absence) of the markers in (automatically identified) regions where the markers’ densities are higher. Hereafter these regions will be referred as “concentration regions”.

From an attentive observation, we noted that each concentration region is generally composed of a core region, where the markers’ density is higher and the pixel distance among markers is less than $\frac{R}{2}$, and a surrounding region, where the markers’ density diminishes and the distance among markers increases until reaching the value *R* on the border of the concentration region. The *R* value changes in each section, but all the concentration regions in the same section are well defined by a unique *R* value. Precisely, given a section *SL*, and denoting its markers with *M*, concentration regions in *SL* are composed by pixels belonging to the tissue region, which are distant less than *R*(*M*) from any marker pixel. To automatically estimate the proper *R*(*M*) we compute the histogram of the minimum distances between each pixel in the tissue region and the markers segmented in *SL*, and we select the value *R*_*MAX*_(*M*) where the histogram reaches its maximum value. If the section does not contain any concentration region, *R*_*MAX*_(*M*) results as too high value. To avoid this problem, we determine the value *R*_*LIMIT*_(*M*); this value is such that the number of pixels at distance less than *R*_*LIMIT*_(*M*) from any marker pixel is less than 50*A*_*M*_, where *A*_*M*_ is the number of marker pixels in *SL*. The value *R*(*M*) is then computed as *R*(*M*) = min(*R*_*LIMIT*_(*M*), *R*_*MAX*_(*M*)).^4^

Having estimated *R*(*M*), we identify core regions by selecting pixels are at a distance less than $\frac{R(M)}{2}$ from any marker pixel, and delete small connected areas (areas with less than $10{\left(\frac{R(M)}{2}\right)}^2$ pixels). The remaining core regions are then expanded to include pixels at a distance less than *R*(*M*) from any marker and the small connected regions (containing less than 20*R*^2^ pixels) are discarded. The remaining connected regions represent the concentration regions in *SL*.

**Fig. 6:**
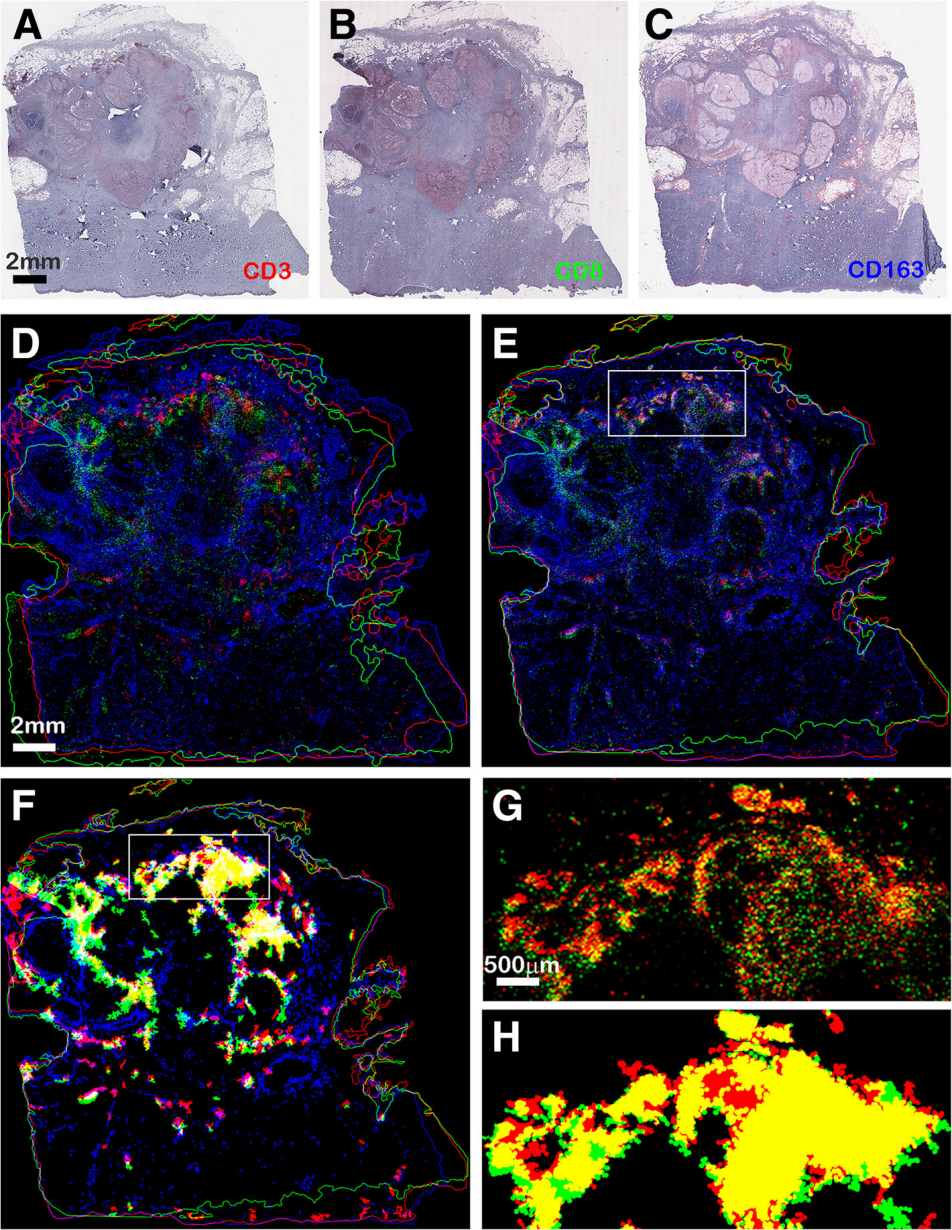
Marker co-existence analysis from serial tissue sections. Top row: metastatic melanoma tissue labelled with CD3 (**a**), CD8 (**b**), and CD163 (**c**) markers stained in reddish color. **d** overlapped tissue shapes before shape-based registration; **e** and overlapped tissue shapes after registration. Note that in **d** the tissue shapes are not aligned; after image registration the tissue shapes are aligned and the co-existing markers are overlapped (as shown in detail **g**). Yellow colors appear when red and green markers are overlapped, purple colors appear when red and blue markers are overlapped, white colors appear when all the three markers are overlapped. **f** overlapped concentration regions after registration; **g** zoomed detail of overlapped CD3 and CD8 markers; **h** zoomed detail of overlapped CD3 and CD8 concentration regions. The GTRO computed on these images before registration equals 76.5%, while it increases to 97.9% after registration

Once concentration regions are found in two sections *SL*_1_ and *SL*_2_, they can be exploited to derive different measures expressing the markers co-existence either in the whole tissue region, in user selected regions of interest (ROIs), such as rectangular areas (Fig. 6e and f), or in selected concentration regions.

As an example, denoting with *M*_1_ and *M*_2_ the markers in two sections *SL*_1_ and *SL*_2_, with *Conc*_1_ and *Conc*_2_ the concentration regions computed by *M*_1_ and *M*_2_, we can compute:

- the density, *DM*_*C*1_ and *DM*_*C*2_, of *M*_1_ and *M*_2_ in their concentration regions; *DM*_*Ci*_ = *A*_*Mi*_/*CA*_*i*_, where *A*_*Mi*_ is the area covered by *M*_*i*_, and *CA*_*i*_ is the area of *Conc*_*i*_, that is the number of pixels composing *Conc*_*i*_;
- the density, *DM*_1*InC*2_ and *DM*_2*InC*1_, of *M*_1_ and *M*_2_ in the concentration regions of the other marker; precisely, ${DM}_{1 InC2}={A}_{M1\bigcap {Conc}_2}/{CA}_2$, ${DM}_{2 InC1}={A}_{M2\bigcap {Conc}_1}/{CA}_1$, where ${A}_{M1\bigcap {Conc}_2}$ is the area of the markers *M*_1_ in *Conc*_2_ and ${A}_{M2\bigcap {Conc}_1}$ is the area of the markers *M*_2_ in *Conc*_1_;
- the weighted mean of *DM*_1*InC*2_ and *DM*_2*InC*1_:${wMean}_{Dens}\left({DM}_{1 InC2},{DM}_{2 InC1}\right)=w\ \frac{DM_{2 InC1}+{DM}_{1 InC2}}{2}$, where $w=\frac{\min \left(\ {DM}_{C1},{DM}_{C2}\right)}{\max \left(\ {DM}_{C1},{DM}_{C2}\right)}$;
- the percentage, *PM*_1*InC*2_ and *PM*_2*InC*1_, of *M*_1_ and *M*_2_ in the concentration regions of the other marker; precisely, ${PM}_{1 InC2}={A}_{M1\bigcap {Conc}_2}/{A}_{M1}$, ${PM}_{2 InC1}={A}_{M2\bigcap {Conc}_1}/{A}_{M2}$.
- the weighted mean of *PM*_1*InC*2_ and *PM*_2*InC*1_: ${wMean}_{AVG}\left({PM}_{1 InC2},{PM}_{2 InC1}\right)=w\ \frac{PM_{2 InC1}+{PM}_{1 InC2}}{2}$.

Though these measures are computed on the whole section, they can be restricted to consider only the markers and concentration regions contained in user-selected ROIs (in this case *DM*_*Ti*_ = *A*_*Mi*_/*ROIA*, where *ROIA* is the area of the user-selected region of interest).

## Results

### Marker segmentation and location analysis

MIAQuant_Learn, our open source software, stands out for its capability to be customized to any marker color appearance thanks to the usage of supervised learning techniques. Of note, its classifiers can be continuously updated by adding training points; this allows increasing their “knowledge” until satisfactory results are computed.

In Fig. 1 (center column) we show three images containing regions whose color, being similar to that of markers, may cause false positive segmentation errors. These are: colorings due to china ink used to identify resection margins (Fig. 1b), stain spread and imprisoned in tissue folds (Fig. 1e), and unspecific colorings in red blood cells (Fig. 1h). Segmentation results computed by MIAQuant_Learn (right column) do not contain the false positive errors computed by MIAQuant (left column). MIAQuant_Learn processed also “old” slides, often biased by color modifications (e.g. by blurring effects and/or by discolorations) and technical deficits. We could obtain successful segmentation results for 98.67% of 1357 images. It must be added that MIAQuant_Learn effectively processes also fluorescence microscopy images, where segmentation problems are easier to overcome.

**Fig. 7:**
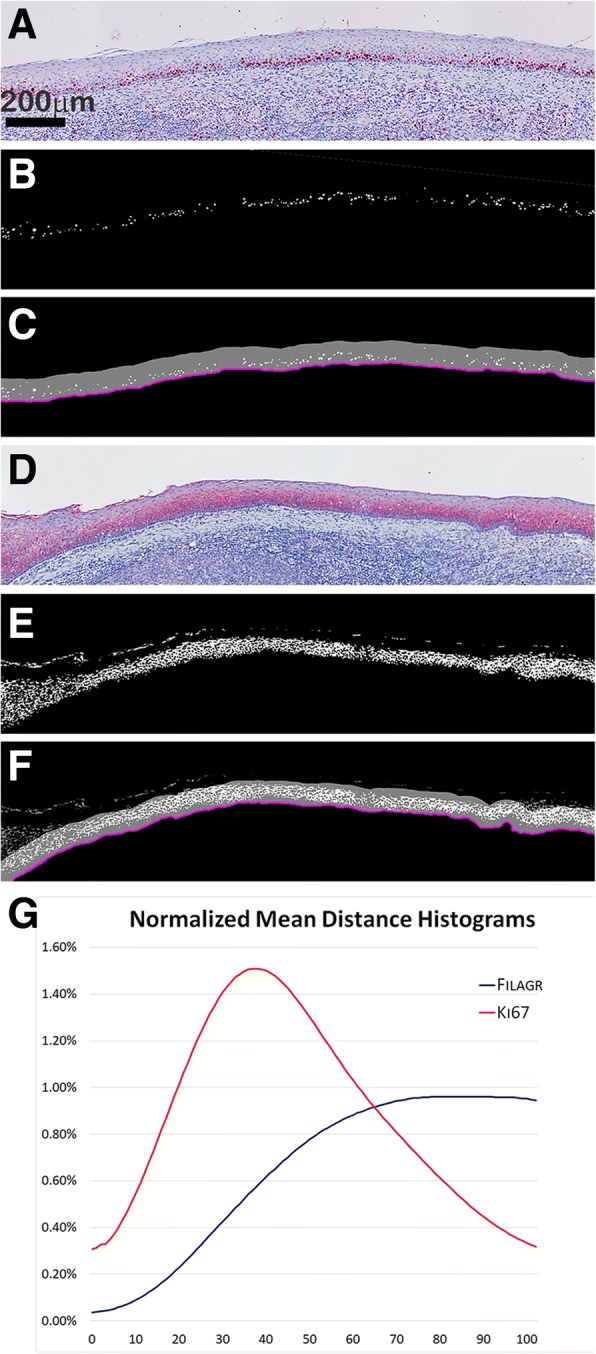
Computation of the mean-distance histograms. **a**, **d** human tonsil sections marked with **a** Ki-67 antibody, and **d** Filagrin antibody (both the antibodies are marked with red stains). **b**, **e** segmented markers. **c**, **f** purple lines showing the manually signed borders of the basement membranes. The gray band shows the border neighborhood considered during the histogram computation; precisely, only markers in the gray band are used to compute the normalized mean-distance histograms plotted in (**g**). The histograms (computed over all the human tonsil dataset) clearly show that the Ki-67 antibody tend to be nearer to the border than the Filagrin one. In this case, the average intersection measure equals 0.60

Once segmented, the markers’ density and (relative) position can be exploited to compute several other measures, such as mean-distance histograms from structures of interest. The histogram plots and the histogram intersection measure allow to visually and numerically assess differences and/or similarities among the markers’ positions. Figure 7 shows two human tonsil sections, belonging to a human tonsil database, stained with Ki-67 and Filagrin antibodies (red) prior to MIAQuant_Learn processing; the sections in database have been studied to understand the distribution of these markers with respect to the basement membrane, manually marked by experts (purple lines in Fig. 7c and Fig. 7f). The mean-distance histograms computed over the whole dataset (plotted in Fig. 7g) confirm the expected marker distribution. Ki-67 marks proliferating cells generally tied to the basement membrane, while Filagrin is contained in differentiating cells, most of which are located far from the basement membrane. The histogram intersection computed by our software equals 0.6, confirming the difference in the markers’ distribution.

This kind of analysis can provide useful information and was applied to analyze the relative location of different cell populations with respect to manually marked borders in arteriosclerotic plaques [69].

### Alignment of serial image sets

To provide visual means for markers’ co-existence detection and analysis, MIAQuant and MIAQuant_Learn automatically align (register) serial sections and compute images where the markers are overlapped. MIAQuant Learn improves the visual information by producing a color image where automatically detected concentration regions (that is regions where each marker density is high) are overlapped.

Note that, since serial section images depict contiguous histological sections whose thickness is similar to, or bigger than, that of the histological structures of interest, when two or more markers are overlapping in the color image computed by MIAQuant_Learn after registration, they must be considered as neighbors rather than adhering. For this reason, the analysis of contiguous sections allows detecting markers co-existing in the same volumes rather than co-localizing markers. Co-localization studies [71, 72] can indeed be performed only on (more expensive) histological images produced from sections contemporaneously stained for different antigens.

The detection of co-existing markers identifying biological structures in volume/areas is relevant to get insight into the complex interactions governing biological processes.

In Fig. 6: we show the result computed by the shape-based registration procedure of MIAQuant Learn on an image set depicting three serial sections of metastatic melanoma marked for CD3 and CD8 lymphocytes and CD163 myeloid cell markers (Fig. 6a-c). The color image computed before registration (Fig. 6d) achieves a *GTRO* value equal to 85.9%, displaying an increase to 93.2% after registration (Fig. 6e), now enabling an objective comparative (visual) analysis of the three markers’ relative position (a detail is shown in Fig. 6g). This confirms the effectiveness of image registration procedure by MIAQuant Learn. Automatically computed (overlapped) concentration regions relative to three markers (CD3, red; CD8, green; CD163, blue) are shown in Fig. 6f. To focus on lymphocytes, we exploited the ability of MIAQuant_Learn to restrict the computation of the co-existence measures in the rectangular ROI shown in Fig. 6e and f (Fig. 6g and h respectively show the overlapped markers and the overlapped concentration regions in the ROI). In Table 1 we show the marker densities in the tissue region, in the ROI, as well as in specific concentration regions. Comparing the computed values reveals that the densities of the three markers in the ROI are higher than those in the whole tissue region, and that they further increase when computed in the automatically extracted concentration regions (Fig. 6h). Importantly, this points out that the three markers have a different increase in density when different areas are considered.

**Table 1:** examples of density measures computed on the sections shown in Fig. 6

Marker name	MARKER DENSITY			
	in tissue region (%)	in rectangular ROI (%)	in CD3’s concentration region (red area in ROI) (%)	In CD8’s concentration region (green area in ROI) (%)
CD3	0.12	0.52	1.28	1.24
CD8	0.13	0.35	0.76	0.88
CD163	1.19	1.56	2.25	2.23

**Fig. 8:**
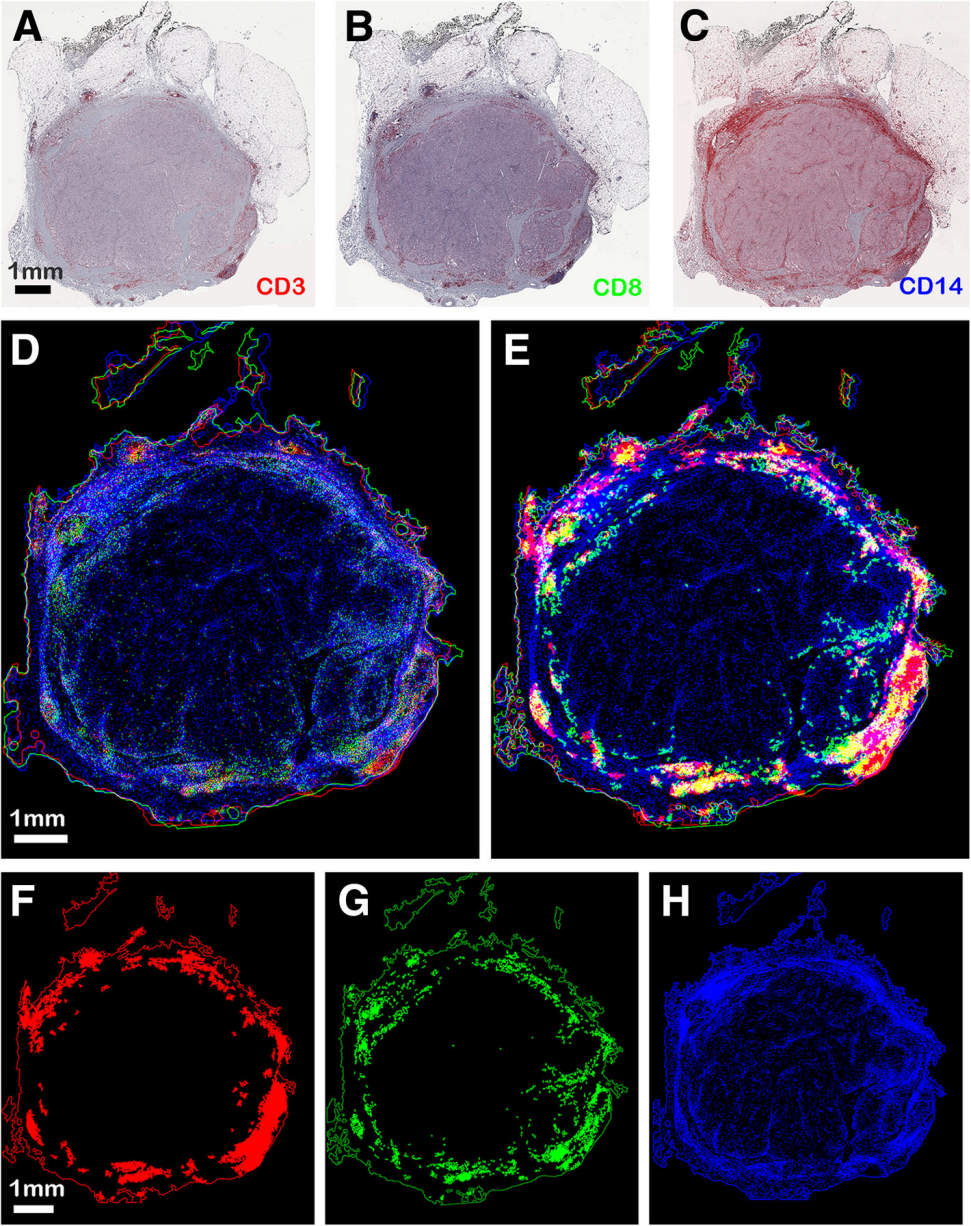
Tissue-based registration of tissue samples and computation of concentration regions. **a-c** metastatic melanoma tissue marked with CD3 (**a**), CD8 (**b**), and CD14 (**c**) antibodies stained in reddish color. **e** overlapped markers after registration; **f** overlapped concentration regions after registration. **f-h** concentration regions of markers CD3 (**f**), CD8 (**g**), and CD14 (**h**). In this case, the GTRO before registration equals 81.2%, while it increases to 97.4% after registration

As a further example, Fig. 8 shows three sections of metastatic melanoma tissue marked with CD3, CD8 and CD14, identifying monocytes antibodies (A-C) and the color image of the overlapped segmented markers after image registration (D). In Fig. 8e the automatically computed concentration regions (Fig. 8f-h) are overlapped. Visual inspection evidences that all three markers are mainly present in the peritumoral area. Table 2 shows that the density values increase when they are computed in concentration regions. Considering that CD14 marks myeloid cells, while CD3 and CD8 markers identify lymphocytes, the comparison of the density values computed in specific areas suggests the potential interaction between these cell populations and allows experts to speculate on their biological function.

**Table 2:** examples of density measures computed on the sections shown in Fig. 8

Marker name	MARKER DENSITY			
	in tissue region (%)	in CD3’s concentration region (red areas) (%)	in CD8’s concentration region (green region) (%)	In CD14’s concentration region (blue region) (%)
CD3	0.94	6.98	4.81	1.98
CD8	1.19	4.31	9.44	2.67
CD14	10.45	25.98	23.70	88.75

A concise way to express the marker co-existence is the computation of the weighted mean of markers’ percentage. Computing this measure on this serial section set we obtained:

$$ {wMean}_{AVG}\left({PM}_{CD3 InCCD8},{PM}_{CD8 InCCD3}\right)=34.06\%; $$

$$ {wMean}_{AVG}\left({PM}_{CD3 InCCD14},{PM}_{CD14 InCCD3}\right)=2.35\%; $$

$$ {wMean}_{AVG}\left({PM}_{CD8 InCCD14},{PM}_{CD14 InCCD8}\right)=2.54\%. $$

These values suggest that the co-existence relationship between marker CD3 and marker CD8 is stronger than those between markers CD3 and CD14, and between markers CD8 and CD14, depending at least in part on the co-expression of CD3 and CD8 by T cells. Despite we here considered as serial section sets those composed of only three, the markers’ co-existence measurements could be computed on sets containing an arbitrary number of serial sections. In this case, the weighted mean of markers’ percentage is a useful measure since it expresses couples of co-existing markers in a unique data.

## Conclusions

In this paper we have described MIAQuant_Learn, a novel system for the automatic segmentation, quantification, and analysis of histological sections acquired by differing techniques and imaging systems. The usage of simple, efficient, and effective image processing, pattern recognition and supervised learning techniques [64], allows any user to customize the marker segmentation to any color appearance. To facilitate the analysis, MIAQuant_Learn computes mean-distance histograms to objectively express the markers’ position and relative location with respect to the resection margins and to user-selected structures of interest. Furthermore, in case of serial tissue sections, MIAQuant_Learn computes objective “morphology-based” measures expressing the markers’ co-existence in areas of higher densities. In the Results section the reported examples show that the introduced system effectively segments and quantifies markers of any color and shape, provides their descriptive analysis, and eventually provide informative measures to help marker co-existence analysis.

Of note, most of the analysis reported in this paper (e.g. in Table 2) was performed on images of high dimension and resolution; obtaining such precision by manual counting procedure would result as exhausting and time-consuming. Moreover, the co-existence analysis provided by MIAQuant_Learn can exploit any serial section set, even those stored long-term in archives for different purposes.

In conclusion, MIAQuant_Learn is reliable, easy to handle and usable even in small laboratories, since image acquisition can be performed by cameras mounted on standard microscopes, which are commonly used in histopathological routine. As flexible, easily modifiable software, it adapts well to meet researchers’ needs and can be applied on different image formats. Due to its potential, MIAQuant_Learn is currently used in several research studies, such as the study of myeloid infiltrate and the definition of immune cell tissue scores in different types of cancer.

MIAQuant_Learn code is available online at www.consorziomia.org for clinical research studies.

### Availability and requirements

Project name: MIAQuant_Learn

Project home page: www.consorziomia.org

Operating system(s): Platform independent

Programming language: Matlab 2017Ra

Other requirements: Matlab 2017Ra

License: Free

Restrictions: No restrictions to use by non-academics.

## Abbreviations

CAD: Computer-Aided Diagnosis
CNN: Convolutional Neural Networks
DT: Decision tree
FRST: Fast Radial Simmetry Transform
GTRO: Global tissue-region overlap
HC: Histochemical
ICA: Independent Component Analysis
IHC: Immunohistochemical
KNN: K Nearest Neighbor
NMF: Non-negative Matrix Factorization
ROI: Region of interest
SVM: Support vector machine

## Footnotes

1 Note that to describe the pixels’ colors we employ features computed in the RGB color space, though other color spaces, e.g. HSV and HIS, might be more intuitive for human perception. However, as reported in [13, 42], this might not be necessarily true for automated classification, whereas colored image segmentation generally achieves better results using the RGB color space; this fact is further confirmed by our statistical analysis [59].

2 The minimum distance between a marker pixel p and a ROI is the distance between p and the pixel in the border of the ROI, which is at the minimum distance from p.

3 The histogram intersection measure is not symmetric. Computing the average allows to obtain a symmetric measure.

4 Note that the value *R*(*M*), estimated for markers *M*, is related to the marker distribution in the tissue region; precisely, given two markers *M*_1_ and *M*_2_ with the same density in the tissue region, values *R*(*M*_1_) >  > *R*(*M*_2_) mean that markers *M*_1_ tend to concentrate in areas were their density is higher, while markers *M*_2_ are more evenly distributed in the whole tissue region.
